# The association between geriatric nutritional risk index and mortality risk of patients after transcatheter aortic valve replacement: a meta-analysis

**DOI:** 10.3389/fnut.2026.1733903

**Published:** 2026-03-20

**Authors:** Xueqian Liu, Yiting Cai, Jiayu Zhang, Shiyu Chen, Shoupei Cao

**Affiliations:** 1 First Clinical Medical College, Nanjing University of Chinese Medicine, Nanjing, China; 2 Department of Cardiology, Nanjing Hospital of Chinese Medicine Affiliated to Nanjing University of Chinese Medicine, Nanjing, China

**Keywords:** aortic stenosis, geriatric nutritional risk index, malnutrition, mortality, transcatheter aortic valve replacement

## Abstract

Malnutrition is common among older adults undergoing transcatheter aortic valve replacement (TAVR) and may adversely affect postoperative survival. The Geriatric Nutritional Risk Index (GNRI), an objective marker of nutritional status, has been proposed as a prognostic tool. However, results from individual studies remain inconsistent. This meta-analysis aimed to clarify the association between pre-procedural GNRI and mortality after TAVR. A systematic search of PubMed, Embase, and Web of Science was conducted to identify observational studies reporting categorical comparisons of GNRI (low vs. high) and all-cause mortality after TAVR. Pooled hazard ratios (HRs) with 95% confidence intervals (CIs) were calculated using random-effects models accounting for the influence of heterogeneity. Thirteen cohort studies involving 9,647 patients were included. The pooled analysis demonstrated that low pre-procedural GNRI was significantly associated with increased all-cause mortality after TAVR (HR = 1.90, 95% CI: 1.60–2.26; *I*^2^ = 43%, *p* < 0.001). A stronger association was observed in prospective studies as compared to retrospective studies (*p* for subgroup difference = 0.04). The association remained robust across subgroups stratified by study country, sample size, GNRI cutoffs, analytical models, and follow-up duration. Meta-regression showed no significant influence of age, sex, body mass index, diabetes prevalence, Society of Thoracic Surgeons (STS) score, or follow-up durations on the effect size. In conclusion, lower GNRI prior to TAVR is associated with a higher risk of all-cause mortality, underscoring the prognostic importance of pre-procedural nutritional assessment. GNRI could be considered as a complementary tool for risk stratification in TAVR candidates, while further prospective studies are needed to determine its optimal clinical integration.

**Systematic review registration:**

The review protocol was prospectively registered in PROSPERO (registration number: CRD420251178097).

## Introduction

Transcatheter aortic valve replacement (TAVR) has revolutionized the management of severe aortic stenosis (AS), offering a less invasive alternative to surgical valve replacement for patients at high or prohibitive surgical risk (1, 2). The global volume of TAVR procedures has increased rapidly, surpassing surgical aortic valve replacement in several developed countries (3, 4). TAVR provides substantial benefits, including improved survival, symptom relief, and functional recovery, particularly in older adults who comprise the majority of the treated population (5). However, despite these advances, post-procedural mortality remains considerable, reflecting the complex interplay of age-related frailty, comorbidities, and procedural complications (6, 7). Conventional risk stratification models, such as the Society of Thoracic Surgeons (STS) score and EuroSCORE II, incorporate demographic and comorbidity variables but often fail to capture geriatric-specific factors such as nutritional and functional status (8, 9). Identifying reliable prognostic markers beyond traditional risk models is therefore crucial to improve patient selection, optimize perioperative management, and enhance long-term outcomes after TAVR.

Malnutrition is common among elderly TAVR candidates and has emerged as an independent predictor of poor prognosis following the procedure (10, 11). The Geriatric Nutritional Risk Index (GNRI)—calculated from serum albumin concentration and the ratio of actual to ideal body weight—provides a simple, objective assessment of nutritional status and overall physiological reserve (12). Importantly, its prognostic utility has also been increasingly recognized in broader cardiovascular settings. For example, the multicenter prospective AFTER-2 study showed that GNRI, alongside controlling nutritional status (CONUT) score and prognostic nutritional index (PNI), independently predicted all-cause mortality in patients with non-valvular atrial fibrillation, supporting the role of nutritional risk assessment in cardiovascular risk stratification beyond valvular interventions (13). Low GNRI reflects protein–energy malnutrition, chronic inflammation, and impaired recovery capacity (12, 14), which may pre-dispose to complications and higher mortality after TAVR. Although several studies have investigated the association between GNRI and post-TAVR outcomes, their results have been inconsistent due to variations in study design, sample size, cutoff definitions, and analytical adjustments (15–27). Moreover, a quantitative summary of the relationship between GNRI and mortality risk, along with an exploration of study-level modifiers, is lacking. Therefore, this meta-analysis was conducted to systematically synthesize the available evidence and clarify the prognostic significance of pre-procedural GNRI in predicting mortality after TAVR.

## Methods

This study was conducted in accordance with the PRISMA 2020 reporting standards (28) and adhered to the methodological recommendations outlined in the Cochrane Handbook for Systematic Reviews and Meta-Analyses (29) throughout the protocol development, data collection, synthesis, and presentation of findings. The review protocol was prospectively registered in PROSPERO (registration number: CRD420251178097).

### Database search

We conducted a systematic literature search in PubMed, Embase, and Web of Science to identify eligible studies using a combination of keywords related to (1) “geriatric nutritional risk index” OR “geriatric nutrition risk index” OR “GNRI” OR “nutritional indices” OR “ nutritional risk index” OR “malnutrition”; and (2) “transcatheter aortic valve implantation” OR “TAVI” OR “transcatheter aortic valve replacement” OR “TAVR”. The search was restricted to human research, full-length original articles written in English, and published in peer-reviewed journals. We also manually reviewed the reference lists of pertinent articles to identify any additional studies. The search covered all available records up to September 22, 2025. Detailed search strategies for each database are provided in Supplementary file 1.

### Study identification

The inclusion criteria were defined using the PICOS framework.

Population (P): adult patients (≥18 years) undergoing TAVR for aortic stenosis or related valvular heart disease.

Intervention/Exposure (*I*): pre-procedural nutritional assessment using GNRI, reported as categorized variables. A low GNRI was considered as malnutrition, with the cutoff values consistent with those in the original studies.

Comparator (C): patients with normal/high GNRI.

Outcomes (O): all-cause mortality during follow-up compared between patients with a low vs. a high GNRI before TAVR.

Study design (S): eligible study types included longitudinal observational designs, such as retrospective and prospective cohort studies, nested case–control analyses, and *post-hoc* evaluations of clinical trial datasets.

We excluded case reports, case series, reviews, editorials, and non-original analyses. Studies not reporting GNRI as an exposure of interest, those without extractable mortality outcomes or insufficient data to calculate effect estimates, and those involving mixed cardiac interventions without distinguishable results for TAVR were also excluded. Animal studies, pediatric studies, and duplicated cohorts were also excluded. In cases where multiple publications used overlapping patient populations, the study with the largest sample size or most complete dataset was retained.

### Study quality evaluation

Two reviewers independently performed the database search, screened eligible studies, assessed methodological quality, and extracted data. Any disagreements were resolved through discussion with the corresponding author. The methodological quality of included studies was appraised using the Newcastle–Ottawa Scale (NOS) (30), which evaluates participant selection, control for confounders, and outcome ascertainment. The scale ranges from 1 to 9 points, with scores ≥7 indicating high methodological quality.

### Data collection

The extracted information included: basic study details (first author, publication year, study design, and country); characteristics of the patients [diagnosis, sample size, mean ages, sex distributions, mean body mass index (BMI) at baseline, proportions of patients with diabetes, and the Society of Thoracic Surgeons risk scores (STS)], exposure features (timing of GNRI measurement, methods for determining the cutoffs of GNRI for malnutrition, cutoff values of GNRI, and number of patients with malnutrition before the procedure), follow-up durations, the number of patients who died during follow-up, and covariates included in adjusted analyses examining the association between pre-operative GNRI and mortality risk after TAVR.

### Statistical analysis

The association between pre-procedural GNRI and the risk of all-cause mortality after TAVR was quantified using hazard ratio (HRs) and their corresponding 95% confidence intervals (CIs), comparing patients with low vs. high GNRI before TAVR. For studies reporting mortality at different follow-up durations, data from the longest available follow-up period were extracted. When multiple effect estimates were presented using different regression models, the most comprehensively adjusted model was selected to ensure the highest level of confounder control. HRs and standard errors were derived either directly from reported CIs or calculated from p-values, followed by logarithmic transformation to stabilize variance and improve normality (29). Between-study heterogeneity was evaluated using the Cochran's Q statistic and the *I*^2^ statistic (31), with *I*^2^ values of < 25%, 25%−75%, and >75% interpreted as low, moderate, and high heterogeneity, respectively. A random-effects model was applied to pool effect sizes, acknowledging clinical and methodological variability across studies (29). Robustness of the pooled estimates was examined through leave-one-out sensitivity analyses (32). Pre-defined subgroup analyses were performed to explore potential effect modifiers, including study design (prospective vs. retrospective), study country (Asian or Western countries), methods for determining the cutoffs of GNRI, cutoff values of GNRI, analytic models (univariate vs. multivariate models), and study quality scores by NOS. For continuous subgrouping variables, median values were used as cutoffs. In addition, a univariate meta-regression analysis was also performed to evaluate the possible impact of study characteristics on the outcome, such as sample size, mean age of the patients, proportion of men, BMI at baseline, proportion of patients with diabetes, STS at baseline, cutoff values of GNRI, and follow-up durations. Potential publication bias was assessed visually with funnel plots and formally using Egger's regression test (33). A two-sided *p*-value < 0.05 was considered statistically significant. All statistical analyses were conducted using RevMan (version 5.3, Cochrane Collaboration, Oxford, UK) and Stata (version 17.0, StataCorp, College Station, TX, USA).

## Results

### Study identification

**Figure 1:**
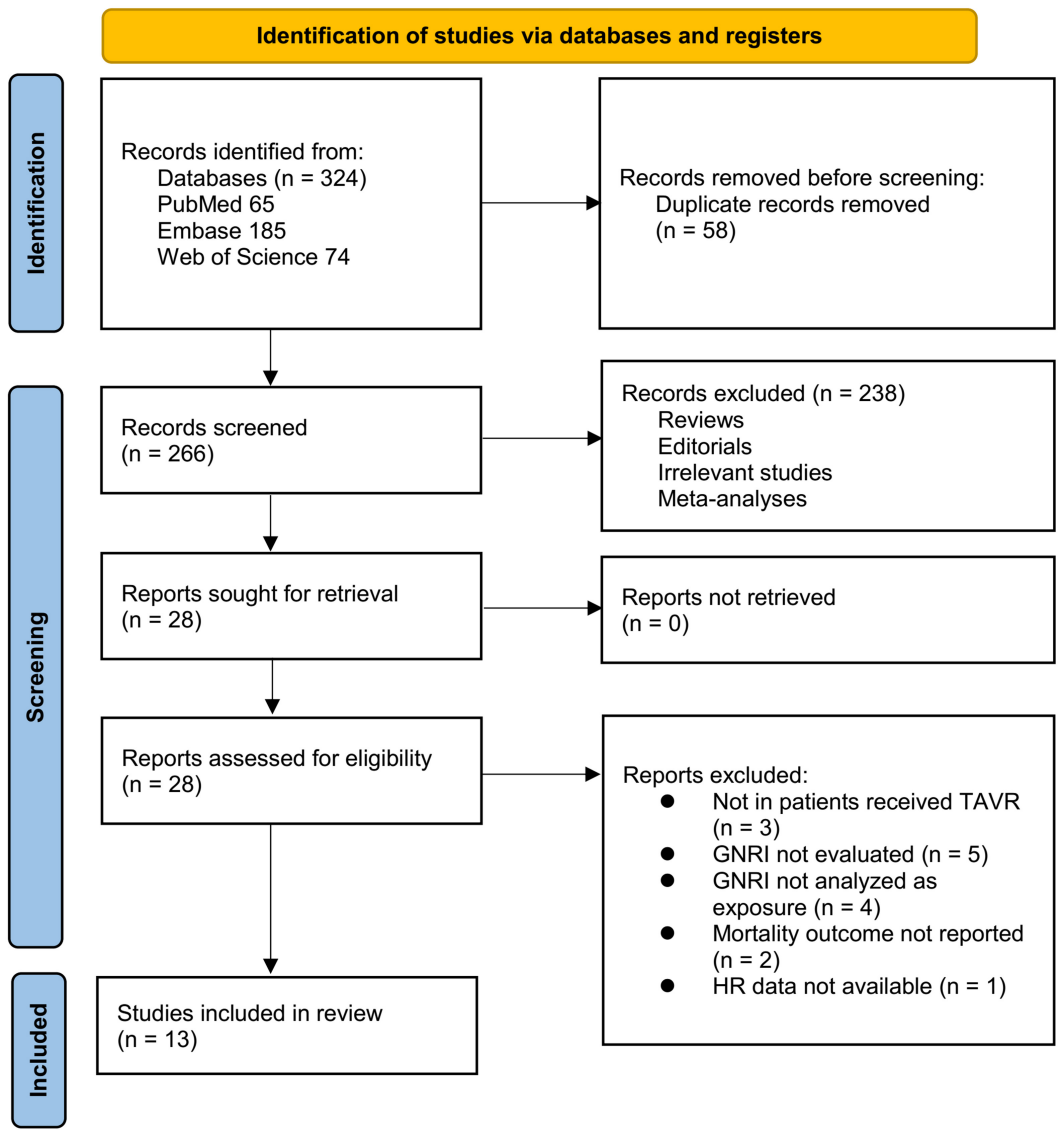
PRISMA 2020 flow diagram of the literature search, study screening, eligibility assessment, and final inclusion process.

Figure 1 outlines the study selection process. Initially, 324 records were identified across three databases, with 58 duplicates removed. After title and abstract screening, 238 articles were excluded for not meeting the meta-analysis criteria. The full texts of the remaining 28 studies were independently reviewed by two authors, leading to the exclusion of 15 for reasons detailed in Figure 1. Ultimately, 13 studies were included in the quantitative analysis (15–27).

### Overview of the study characteristics

Table 1 summarizes the characteristics of the 13 studies included in this meta-analysis, comprising five prospective cohort (15, 21, 22, 24, 25) and seven retrospective cohort studies (16–20, 23, 26, 27) conducted between 2018 and 2024 across Japan, Spain, the United States, South Korea, Germany, Turkey, Italy, and China. All studies enrolled adults with severe or symptomatic aortic stenosis undergoing TAVR. A total of 9,647 patients were included, with sample sizes ranging from 95 to 1,985 participants. The mean age of participants varied from 72.0 to 85.0 years, and the proportion of men ranged between 29.5 and 59.6%. Mean BMI values were 21.8–28.7 kg/m^2^, and the prevalence of diabetes ranged from 12.3 to 42.5%. The STS risk score ranged from 2.4 to 8.9%, reflecting moderate operative risk in most cohorts. The GNRI was consistently measured pre-operatively, typically on admission or within one day before TAVR. GNRI cutoff values used to define malnutrition varied from 91.8 to 114.7, with most derived from previous literature (15–17, 19, 23–27) and some determined through receiver operating characteristic (ROC) curve analysis (18, 21) or median values (20, 22). Accordingly, a total of 4,339 (45.0%) patients were evaluated to be with malnutrition as evidenced by a low GNRI before TAVR. Follow-up durations ranged from approximately 9.4 to 32.2 months (mean: 17.4 months). All studies assessed all-cause mortality as the primary endpoint, and 11 of the included studies performed multivariable-adjusted analyses, controlling for key confounders such as age, sex, BMI, STS score, comorbidities (e.g., diabetes, chronic kidney disease, chronic obstructive pulmonary disease, heart failure), left ventricular ejection fraction (LVEF), and procedural approach to a varying degree (15–17, 19–21, 23–27). Only crude HR data were available in the other two studies (18, 22).

**Table 1:** Characteristics of the included studies.

**Study**	**Design**	**Country**	**Diagnosis**	**No. of patientsincluded**	**Mean age (years)**	**Men (%)**	**Mean BMI (kg/m^2^)**	**DM (%)**	**Mean STS score, %**	**Timing of GNRImeasurement**	**Methods for defining cutoff for GNRI**	**Cutoff of GNRI formalnutrition**	**No. of patients withmalnutrition**	**Follow-up duration (months)**	**No. of patients died**	**Variables adjusted**
Shibata 2018	PC	Japan	Severe AS undergoing TAVR	1,613	84.5	29.6	21.8	26.6	8.9	Pre-operative	Previous study derived	92	528	9.4	174	Age, sex, BMI, STS score, NYHA class III/IV, BNP, SCr, Hb, PAD, Prior CABG, pulmonary disease, liver disease, and transfemoral access
Ferreiro 2019	RC	Spain	Severe AS undergoing TAVR	941	80.7	43.4	28.6	33.9	5.8	Pre-operative (The day before TAVR)	Previous study derived	97.5	453	25.2	186	Age, PAD, prior HF, COPD, CKD, Hb, STS score, femoral approach, Post-TAVR aortic regurgitation grade III-IV, In-hospital major bleeding, and need of pacemaker
Okuno 2019	RC	Japan	Severe AS undergoing TAVR	95	84.0	29.5	21.9	23.2	5.2	Pre-operative (on admission)	ROC curve analysis	91.8	41	12.0	9	None
Saric 2019	RC	USA	Severe AS undergoing TAVR	567	81.2	52.7	28.7	42.5	8.2	Pre-operative	Previous study derived	98	417	8.6	NR	Age, STS score, dialysis, dyslipidemia, cirrhosis, pre-existing AVB, and NYHA class
Lee 2019	RC	South Korea	Severe AS undergoing TAVR	412	78.7	48.1	23.9	31.8	3.2	Pre-operative	Previous study derived	98	227	16.4	35	Age, sex, diabetes, prior MI/PAD/PCI, renal insufficiency, dialysis, transfemoral approach, Hb, Vmax, LVEF, STS score, and RBBB
Mas-Peiro 2021	PC	Germany	Severe AS undergoing TAVR	114	82.8	59.6	27.2	12.3	NR	Pre-operative (on admission)	Median	114.7	57	12.0	23	None
Kucukosmanoglu 2021	PC	Turkey	Severe symptomatic AS undergoing TAVR	119	77.1	41.3	27.3	29.4	7.6	Pre-operative (on admission)	ROC curve analysis	102.5	40	12.0	31	Age, sex, MI, albumin, EDV, Total Cholesterol, BNP, Logistic EuroSCORE, and STS score
Koseki 2021	RC	USA	Severe AS undergoing TAVR	968	82.1	58.0	27.2	32.0	4.8	Pre-operative	Median	103	517	24.0	165	Age, STS score, renal insufficiency, COPD, LVEF, moderate-to-severe MR, and moderate-to-severe TR
Seoudy 2021	RC	Germany	Severe symptomatic AS undergoing transfemoral TAVR	953	82.1	45.4	26.1	29.2	NR	Pre-operative (on admission)	Previous study derived	98	335	21.1	NR	Age, albumin, hs-TNT, NT-proBNP, life-threatening bleeding, AKIN Stage 3, disabling stroke, logistic EuroSCORE, TR III-IV, and PAH
Massussi 2022	PC	Italy	Severe symptomatic AS undergoing TAVR	1,985	81.5	44.7	26.7	28.1	NR	Pre-operative	Previous study derived	98	731	12.0	198	Age, Female, EuroSCORE II, COPD, liver failure, active malignancy, DM, AF, eGFR, previous cardiac surgery, neurological dysfunction, LVEF < 50%, sPAP ≥60mmHg, and NYHA class
Vivendar 2022	RC	China	AS undergoing TAVR	285	79.2	53.7	22.3	18.1	7.8	Pre-operative	Previous study derived	98	79	12.0	21	Age, STS score, hypertension, and diabetes
Ishizu 2022	PC	Japan	Severe symptomatic AS undergoing TAVR	1040	85.0	32.9	22.0	22.0	6.0	Pre-operative	Previous study derived	97.5	629	32.2	274	Age, sex, BMI, Clinical Frailty Scale, NYHA class III/IV, STS-PROM score, dyslipidemia, AF, PAD, active cancer, Hb, eGFR, BNP, LVEF, moderate to severe MR, transfemoral approach, and moderate to severe LVOT calcification
Yang 2024	RC	China	Severe symptomatic AS undergoing TAVR	555	72.0	57.7	23.0	21.3	2.4	Pre-operative	Previous study derived	98	285	17.0	51	Age, NYHA class ≥III, AST, NT-proBNP, LVEF, mean gradient, moderate/severe TR, and PAH

PC, prospective cohort; RC, retrospective cohort; AS, aortic stenosis; TAVR, transcatheter aortic valve replacement; BMI, body mass index; DM, diabetes mellitus; STS, society of thoracic surgeons risk score; GNRI, geriatric nutritional risk index; ROC, receiver operating characteristic; NR, not reported; BNP, B-type natriuretic peptide; SCr, serum creatinine; Hb, hemoglobin; PAD, peripheral artery disease; CABG, coronary artery bypass grafting; HF, heart failure; COPD, chronic obstructive pulmonary disease; CKD, chronic kidney disease; PCI, percutaneous coronary intervention; AVB, atrioventricular block; RBBB, right bundle branch block; Vmax, peak aortic jet velocity; LVEF, left ventricular ejection fraction; MR, mitral regurgitation; TR, tricuspid regurgitation; hs-TNT, high-sensitivity troponin T; NT-proBNP, N-terminal pro-B-type natriuretic peptide; AKIN, Acute Kidney Injury Network stage; PAH, pulmonary arterial hypertension; EuroSCORE II, European system for cardiac operative risk evaluation II; AF, atrial fibrillation; eGFR, estimated glomerular filtration rate; sPAP, systolic pulmonary artery pressure; LVOT, left ventricular outflow tract; EDV, end-diastolic volume.

### Study quality evaluation

As presented in Table 2, study quality was assessed using the NOS. The total scores ranged from 7 to 9, suggesting that all included studies were of high methodological quality. Eight studies achieved the highest score of 9 (16, 17, 20, 21, 23–25, 27), reflecting representative cohorts, appropriate control of key confounders (age and other clinical factors), reliable outcome ascertainment, and adequate follow-up duration of ≥1 year. Five studies scored 7 (18, 19, 22) or 8 (15, 26), mainly due to partial adjustment for covariates or limited follow-up completeness. Importantly, all studies used validated measures of GNRI and provided clear definitions of mortality outcomes. Overall, the methodological rigor of the included studies supports a high level of confidence in the pooled estimates assessing the association between low GNRI and increased mortality risk after TAVR.

**Table 2:** Study quality evaluation via the Newcastle-Ottawa scale.

**Study**	**Representativeness of the exposed cohort**	**Selection of the non-exposed cohort**	**Ascertainment of exposure**	**Outcome not present at baseline**	**Control for age**	**Control for other confounding factors**	**Assessment of outcome**	**Enough long follow-up duration**	**Adequacy of follow-up of cohort**	**Total**
Shibata 2018	1	1	1	1	1	1	1	0	1	8
Ferreiro 2019	1	1	1	1	1	1	1	1	1	9
Okuno 2019	1	1	1	1	0	0	1	1	1	7
Saric 2019	0	1	1	1	1	1	1	0	1	7
Lee 2019	1	1	1	1	1	1	1	1	1	9
Mas-Peiro 2021	1	1	1	1	0	0	1	1	1	7
Kucukosmanoglu 2021	1	1	1	1	1	1	1	1	1	9
Koseki 2021	1	1	1	1	1	1	1	1	1	9
Seoudy 2021	1	1	1	1	1	1	1	1	1	9
Massussi 2022	1	1	1	1	1	1	1	1	1	9
Vivendar 2022	0	1	1	1	1	1	1	1	1	8
Ishizu 2022	1	1	1	1	1	1	1	1	1	9
Yang 2024	1	1	1	1	1	1	1	1	1	9

### Meta-analysis results

**Figure 2:**
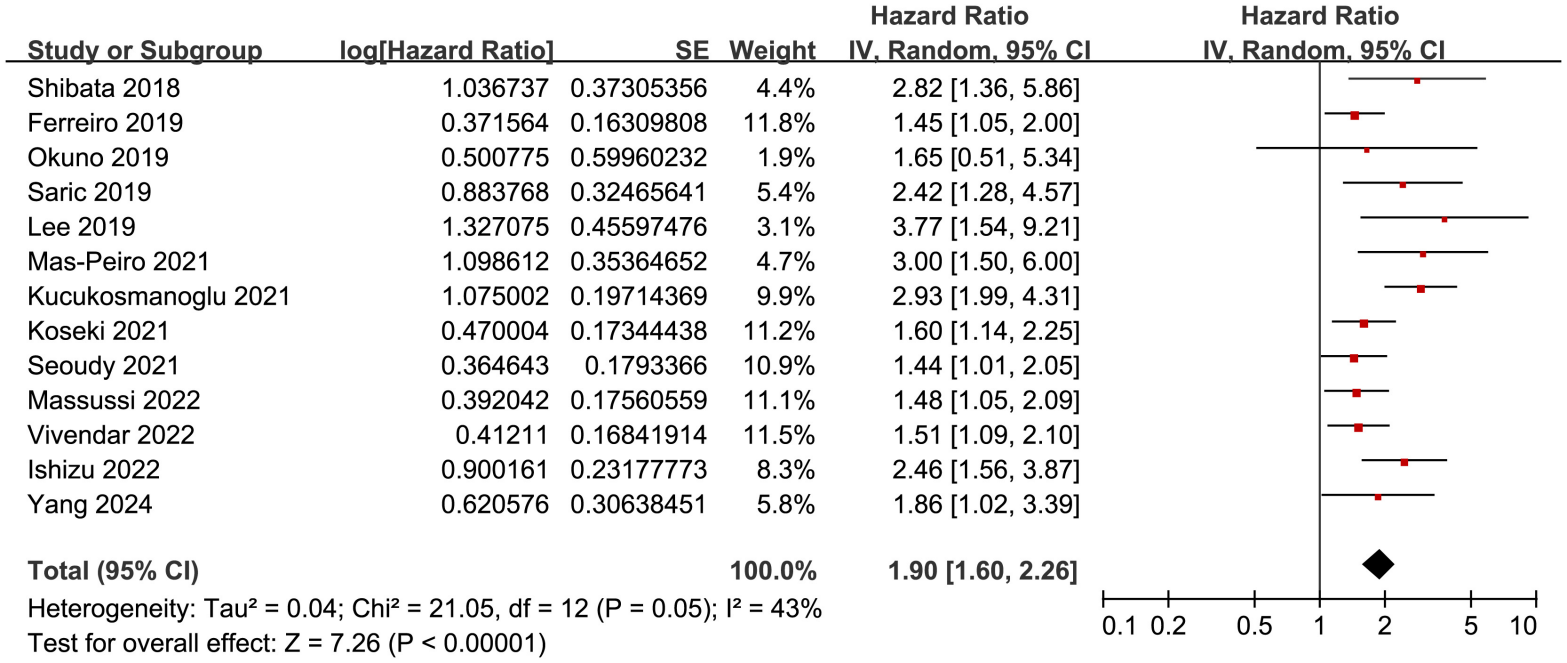
Forest plots for the meta-analysis of the association between pre-procedural GNRI and the risk of mortality after TAVR.

The pooled results of the 13 cohort studies (15–27) showed that overall, malnutrition as evidenced by pre-procedural GNRI was associated with an increased risk of mortality after TAVR (HR: 1.90, 95% CI: 1.60 to 2.26, *p* < 0.001) with moderate heterogeneity (*p* for Cochran's Q test = 0.05; I^2^ = 43%; Figure 2). Sensitivity analysis by excluding one study at a time showed consistent results (HR: 1.76 to 1.97, *p* all < 0.05).

**Figure 3:**
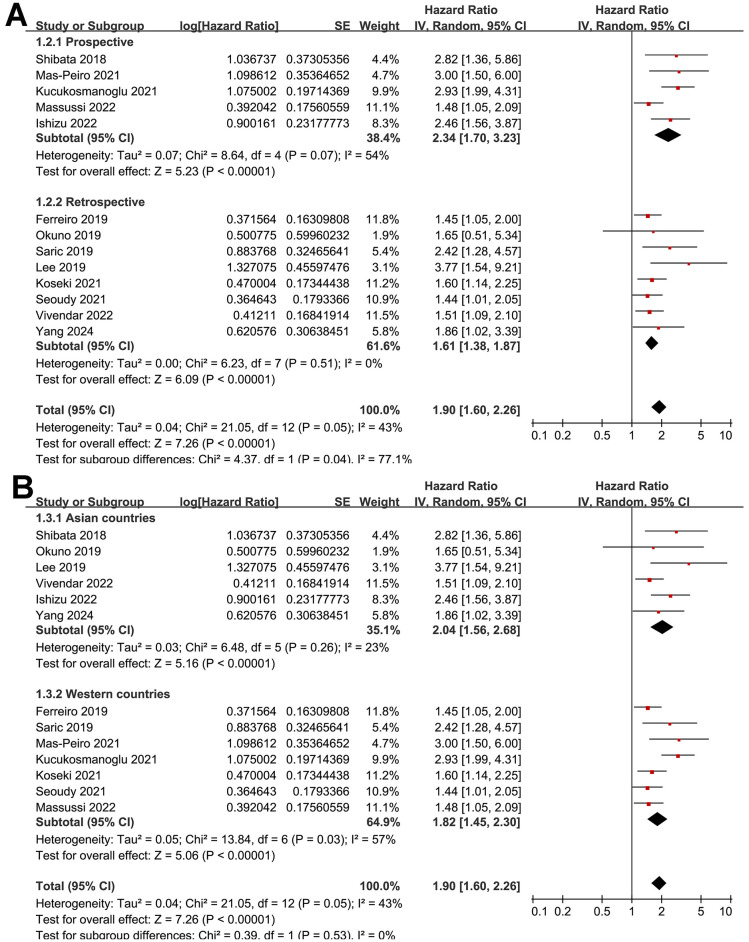
Forest plots for the subgroup analysis of the association between pre-procedural GNRI and the risk of mortality after TAVR; **(A)** subgroup analysis according to the study design; and **(B)** subgroup analysis according to the study country.

**Figure 4:**
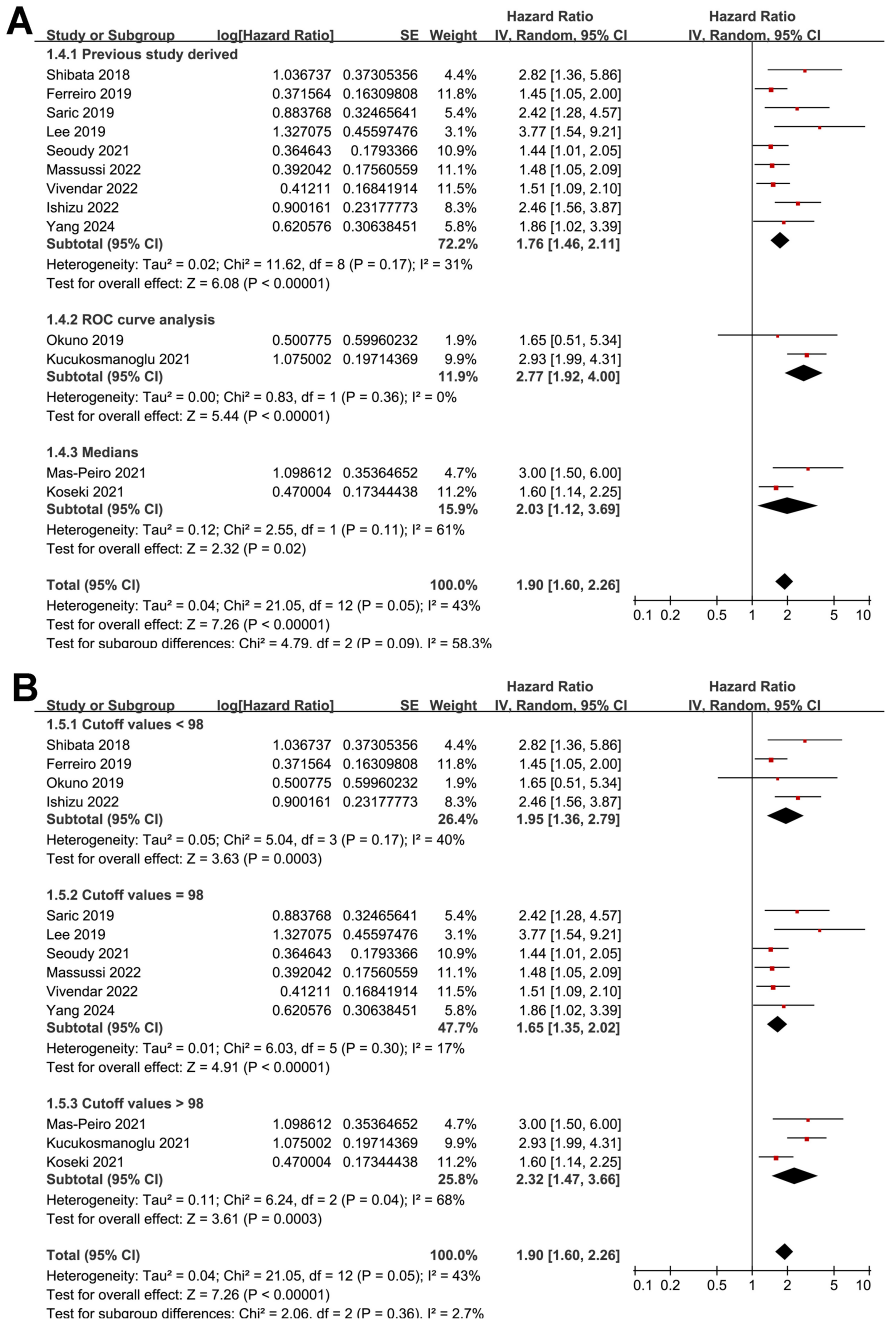
Forest plots for the subgroup analysis of the association between pre-procedural GNRI and the risk of mortality after TAVR; **(A)** subgroup analysis according to the methods for determining the cutoffs of GNRI for malnutrition; and **(B)** subgroup analysis according to the cutoff values of GNRI for malnutrition.

**Figure 5:**
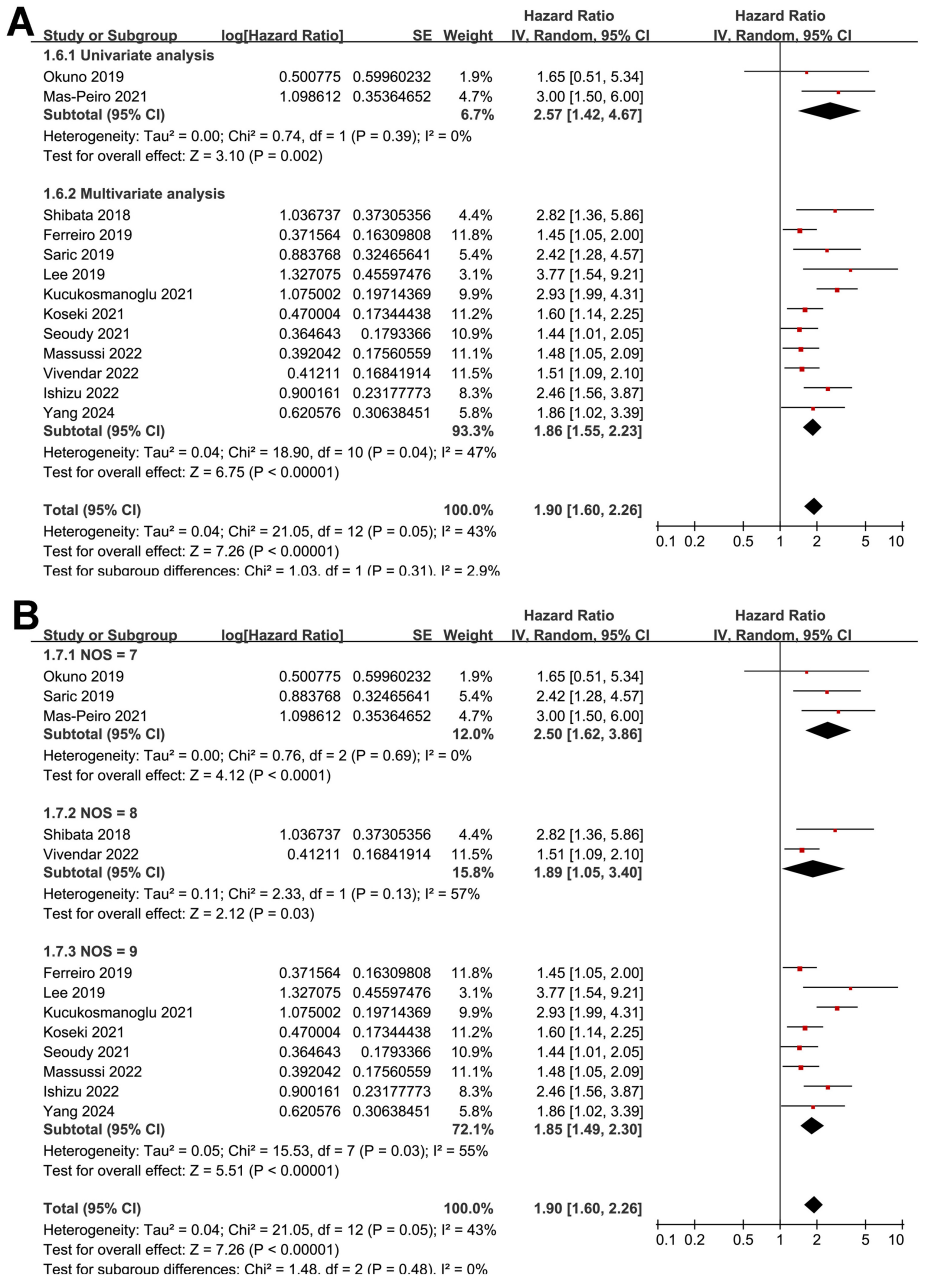
Forest plots for the subgroup analysis of the association between pre-procedural GNRI and the risk of mortality after TAVR; **(A)** subgroup analysis according to the methods for determining the analytic models; and **(B)** subgroup analysis according to the study quality scores via NOS.

Further subgroup analysis showed a stronger association between low GNRI and increased risk of mortality in prospective cohorts compared to retrospective cohorts (HR: 2.34 vs. 1.61, *p* for subgroup difference = 0.04; Figure 3A). Similar results were obtained in studies from Asian and Western countries (HR: 2.04 vs. 1.82, *p* for subgroup difference = 0.53; Figure 3B). Further subgroup analyses showed that the association between a low GNRI and the increased risk of mortality was consistent in studies with the cutoff of GNRI derived from previous studies, ROC curve analysis, or medians of GNRI (HR: 1.76, 2.77 vs. 1.90, *p* for subgroup difference = 0.09; Figure 4A), and in studies with cutoff values of GNRI < 98, = 98, or >98 (HR: 1.95, 1.65 vs. 2.32, *p* for subgroup difference = 0.36; Figure 4B). Moreover, the results were not statistically significant between studies with univariate and multivariate analyses (HR: 2.57 vs. 1.86, *p* for subgroup difference = 0.31; Figure 5A), or among studies with the NOS of 7, 8, and 9 (HR: 2.50, 1.89 vs. 1.85, *p* for subgroup difference = 0.48; Figure 5B).

Finally, results of the univariate meta-regression analyses suggested that none of the following covariates was significantly correlated with the HR of the association between the low GNRI and increased risk of mortality after TAVR, including study sample size, mean patient age, proportion of men, mean BMI, proportion of the diabetic patients, mean STS risk scores, cutoff values of GNRI, or the follow-up durations (*p* all > 0.05; Table 3).

**Table 3:** Results of univariate meta-regression analysis.

**Variables**	HR for the association between GNRI and mortality of patients after TAVR			
	**Coefficient**	**95% CI**	***p*** **-values**	**Adjusted** *R*^2^
Sample size	−0.00021	−0.00055 to 0.00013	0.21	17.6%
Mean age (years)	−0.0032	−0.0707 to 0.0642	0.92	0%
Men (%)	−0.0078	−0.0305 to 0.0149	0.46	1.3%
Mean BMI (kg/m^2^)	−0.017	−0.100 to 0.067	0.67	0%
DM (%)	−0.0038	−0.0339 to 0.0262	0.78	0%
Mean STS (%)	0.027	−0.112 to 0.166	0.66	0%
Cutoff of GNRI	0.020	−0.025 to 0.065	0.34	9.7%
Follow-up duration (months)	−0.0098	−0.0389 to 0.0193	0.47	0%

HR, hazard ratio; GNRI, geriatric nutritional risk index; TAVR, transcatheter aortic valve replacement; CI, confidence interval; BMI, body mass index; DM, diabetes mellitus; STS, society of thoracic surgeons risk score.

### Publication bias

**Figure 6:**
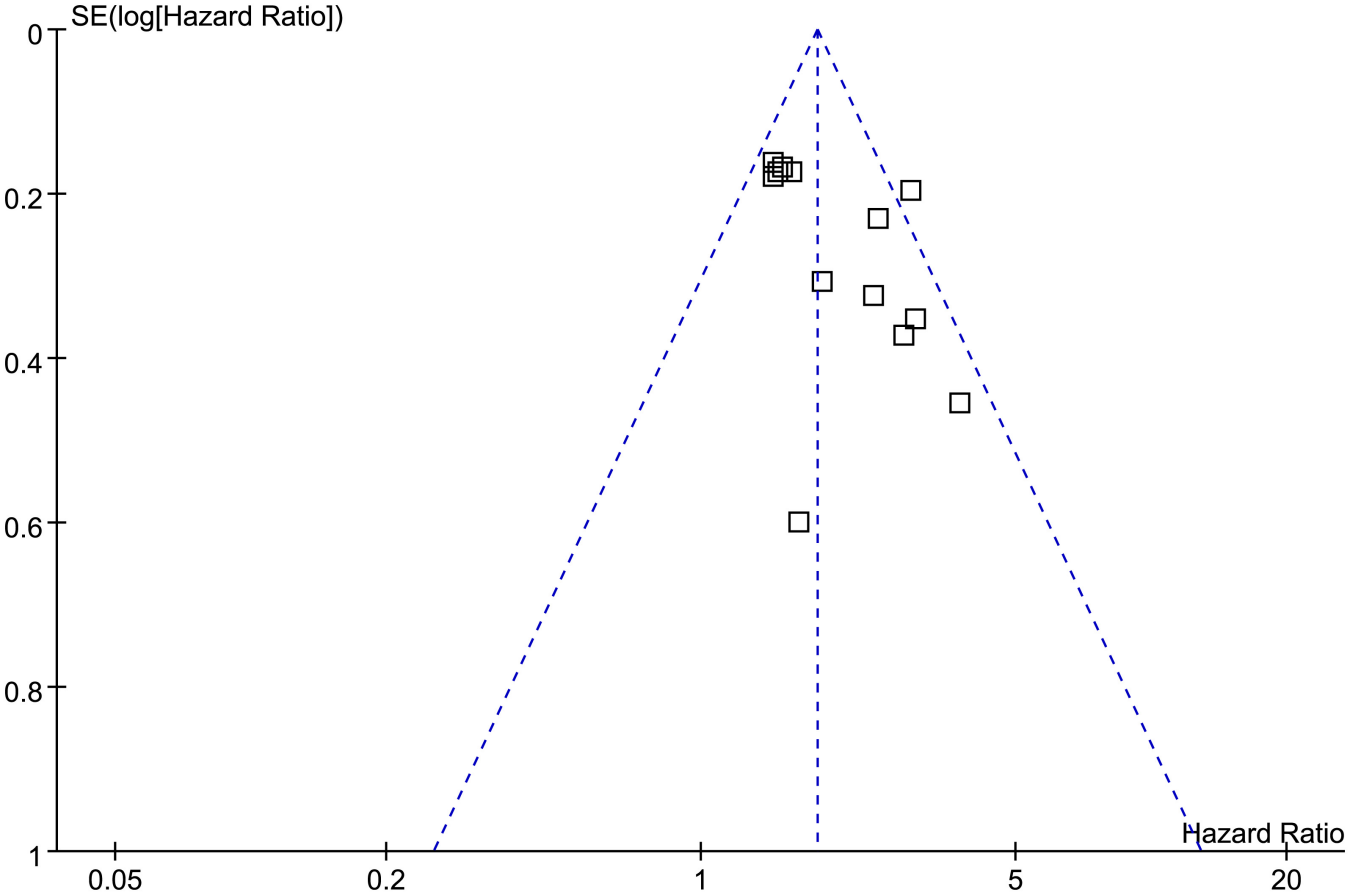
Funnel plots for evaluating the potential publication bias underlying the meta-analysis of the association between pre-procedural GNRI and the risk of mortality after TAVR.

Figure 6 displays the funnel plots for the meta-analysis of the association between the low GNRI and increased risk of mortality after TAVR. The funnel plots are symmetrical on visual inspection, suggesting a low risk of publication bias. The findings are further supported by Egger's regression analysis, which also did not suggest a significant publication bias (*p* = 0.29).

## Discussion

The present meta-analysis provides comprehensive and quantitative evidence that lower pre-procedural GNRI is associated with an increased risk of all-cause mortality in patients undergoing TAVR. These findings underscore the prognostic value of nutritional status, a frequently overlooked but modifiable factor, in an elderly and comorbidity-burdened population. The pooled results across 13 studies involving 9,647 patients demonstrated that low GNRI nearly doubles the risk of post-procedural mortality, supporting the hypothesis that malnutrition reflects impaired physiological reserve, chronic inflammation, and vulnerability to procedural stress. This association remained consistent across diverse populations, study designs, and analytical models, suggesting that GNRI captures important biological domains not incorporated into traditional risk scores such as the STS score or EuroSCORE II.

Several plausible mechanisms may explain the relationship between low GNRI and adverse outcomes following TAVR. GNRI combines serum albumin concentration and body weight relative to ideal weight, both of which represent nutritional and inflammatory status (34, 35). Serum albumin serves not only as a marker of protein–energy malnutrition but also reflects systemic inflammation, hepatic synthetic function, microvascular integrity, and overall metabolic reserve (36, 37). Beyond being a nutritional surrogate, hypoalbuminemia has important pathophysiological consequences, including reduced plasma oncotic pressure, impaired endothelial stability, and altered drug binding and transport, all of which may pre-dispose patients to fluid imbalance, peripheral congestion, and hemodynamic vulnerability after TAVR (38, 39). In addition, low albumin is closely linked to chronic inflammatory activation and immune dysregulation, which may increase susceptibility to postoperative infection and delay tissue repair (40). Malnutrition is also frequently accompanied by sarcopenia and frailty, leading to diminished cardiopulmonary reserve, reduced exercise tolerance, and impaired rehabilitation capacity in elderly patients undergoing transcatheter intervention (41). Similarly, low body weight or weight loss indicates loss of muscle mass and sarcopenia, leading to reduced exercise tolerance, impaired immune defense, and delayed rehabilitation (42–44). Together, these components reflect the biological frailty and diminished homeostatic capacity that make malnourished individuals less capable of recovering from procedural and hemodynamic stress (45). Moreover, inflammation and protein depletion may exacerbate myocardial dysfunction and impair endothelial repair, further contributing to poor long-term survival (46).

The consistency of the findings across subgroups reinforces the robustness of the observed association. The stronger relationship between GNRI and mortality observed in prospective cohorts compared with retrospective studies may reflect better data quality, more consistent follow-up, and more comprehensive adjustment for confounding in prospective designs. Subgroup analyses stratified by study region, sample size, cutoff definitions, and analytical models revealed stable effect sizes, supporting the generalizability of GNRI across populations with varying ethnic and clinical backgrounds. Meta-regression further demonstrated that the prognostic impact of GNRI was independent of conventional risk factors such as age, sex, body mass index, diabetes prevalence, and STS score. These findings highlight that GNRI provides incremental prognostic information beyond established surgical and procedural risk scores. Sensitivity analyses confirmed the stability of the results, and the symmetrical funnel plot suggested no major publication bias. Together, these results indicate a strong and reproducible link between pre-procedural malnutrition and mortality after TAVR.

This meta-analysis has several notable strengths. First, it represents the most up-to-date and comprehensive quantitative synthesis of the evidence on GNRI and post-TAVR mortality. All included studies were observational with longitudinal follow-up, ensuring appropriate temporal assessment between exposure and outcome. Second, the inclusion of multiple subgroup, sensitivity, and meta-regression analyses enhances the robustness and interpretability of the findings. Finally, the studies collectively encompass 9,647 patients from diverse geographic regions and healthcare systems, improving external validity. The use of a random-effects model accounted for between-study heterogeneity, ensuring that the pooled estimate reflects an average effect across different study populations and settings.

Nonetheless, several limitations should be acknowledged. A proportion of the included studies were retrospective in nature, introducing potential selection bias and incomplete control for confounding factors. Retrospective data collection may also be subject to recall and documentation bias, particularly regarding comorbidities and laboratory measurements (47). Although we used multivariable-adjusted HRs whenever available, residual confounding cannot be fully excluded. Patient populations varied across studies in terms of baseline characteristics, nutritional profiles, and procedural approaches, which may have contributed to heterogeneity. Institutional experience and operator volume are well-recognized determinants of TAVR safety and effectiveness (48). However, these variables were not consistently reported in the included cohorts and therefore could not be examined as potential effect modifiers. This limitation may have contributed to between-study variability and restricts our ability to account for important center-level influences on mortality outcomes. Furthermore, the follow-up duration in most studies was relatively short (approximately 1–2 years), which may not fully capture long-term mortality trends. Notably, GNRI cutoff values varied widely across studies (ranging from 91.8 to 114.7), suggesting that the definition of ‘low GNRI' may capture clinically distinct nutritional risk profiles in different cohorts, which could introduce classification heterogeneity and limit the standardization of risk thresholds for clinical application. Moreover, nutritional habits and baseline dietary patterns vary across populations, which may influence the distribution of GNRI values and the applicability of specific cutoff thresholds in different geographic or cultural settings. In addition, key geriatric domains such as frailty (49), cognitive impairment (50), and physical function (51) were rarely assessed in parallel with GNRI, limiting our ability to evaluate their combined prognostic impact. Importantly, as all included studies were observational, causality cannot be inferred, and interventional evidence to confirm that improving GNRI leads to better survival remains lacking. Additionally, because our search was limited to full-text articles published in English, language bias cannot be excluded, and relevant evidence reported in non-English publications may have been missed. Although publication bias was not detected, the possibility of selective reporting of positive findings cannot be entirely ruled out. Finally, because GNRI incorporates body weight relative to ideal weight, its interpretation may be less precise in individuals with obesity, where excess adiposity may not accurately represent nutritional or functional reserve. Future studies may consider whether modified indices incorporating body composition could improve risk stratification.

Several nutritional indices have been proposed for cardiovascular risk stratification, including the CONUT score and the PNI. These tools incorporate different biological domains, such as lymphocyte count and cholesterol levels (CONUT) or immune–nutritional balance (PNI), whereas GNRI is derived solely from serum albumin and body weight relative to ideal weight, making it particularly practical and objective in routine geriatric care (34). Quantitatively, the multicenter prospective AFTER-2 study in non-valvular atrial fibrillation demonstrated that GNRI, CONUT, and PNI were all independently associated with mortality, although their discriminative ability was only modest (13), suggesting that nutritional indices may provide complementary rather than interchangeable prognostic information. In the TAVR setting, GNRI may be especially attractive because it relies on universally available parameters and reflects both protein–energy malnutrition and physiological reserve, which are highly relevant in elderly patients undergoing transcatheter intervention (52).

From a clinical perspective, the findings of this study emphasize the importance of incorporating nutritional assessment into the pre-procedural evaluation of TAVR candidates. The GNRI offers several practical advantages compared with other nutritional or frailty indices: it requires only serum albumin and body weight data, both routinely available, making it simple, cost-effective, and objective (53). In contrast to more complex tools such as the Mini Nutritional Assessment or subjective global assessment, GNRI provides a quantitative and reproducible measure of nutritional risk that can be readily implemented in clinical workflows (54). From a practical standpoint, GNRI may be incorporated as an adjunct to conventional procedural risk scores rather than a replacement. For example, STS or EuroSCORE II could be used as the initial step to estimate surgical and procedural risk, followed by GNRI-based nutritional screening to capture geriatric vulnerability and physiological reserve. Patients with both high procedural risk and low GNRI may represent a subgroup requiring intensified multidisciplinary evaluation, closer peri-procedural monitoring, and consideration of supportive strategies such as nutritional assessment and rehabilitation planning. Importantly, such an approach should be viewed as a conceptual framework, and future prospective research is warranted to determine whether GNRI-enhanced models improve discrimination and clinical decision-making beyond existing scoring systems. Moreover, unlike traditional surgical risk scores that rely primarily on comorbidities and procedural variables, GNRI captures systemic health and physiological reserve, complementing existing models (55). Clinicians should consider assessing GNRI as part of a multimodal risk evaluation, particularly in elderly or frail patients who may otherwise appear low risk by conventional scores. The clinical implications of this finding are two fold. First, identifying patients with low GNRI before TAVR may facilitate closer monitoring and consideration of supportive strategies, but evidence is still needed to determine whether pre-procedural nutritional optimization can modify prognosis. Second, emerging evidence suggests that improvements in GNRI after TAVR are associated with better long-term survival and fewer heart failure hospitalizations (56). This implies that nutritional status is not only a prognostic marker but also a potentially modifiable risk factor. Future research should focus on interventional studies aimed at optimizing nutrition before and after TAVR to determine whether improving GNRI can translate into improved clinical outcomes. Additionally, integrating GNRI with other geriatric assessments—such as frailty indices, sarcopenia measures, and inflammatory biomarkers—may yield more comprehensive risk prediction models.

## Conclusions

In conclusion, this meta-analysis demonstrates that a lower pre-procedural GNRI is associated with increased all-cause mortality after TAVR. These results highlight the critical role of nutritional status as a key determinant of prognosis in patients with severe aortic stenosis undergoing transcatheter intervention. Given its simplicity, objectivity, and prognostic association with mortality, GNRI could be considered as a complementary component of pre-TAVR risk assessment, alongside existing clinical and surgical models, pending further prospective validation. Routine nutritional screening may help identify patients at higher risk of adverse outcomes after TAVR, although whether nutritional interventions can directly improve recovery or survival requires confirmation in prospective interventional studies.

## Data Availability

The original contributions presented in the study are included in the article/Supplementary material, further inquiries can be directed to the corresponding author.
