# Supplementary material for: The association between geriatric nutritional risk index and mortality risk of patients after transcatheter aortic valve replacement: a meta-analysis
Source: Front Nutr. 2026 Mar 20;13:1733903. doi: 10.3389/fnut.2026.1733903 (PMC13046511; doi:10.3389/fnut.2026.1733903)
Supplement: Supplementary file 1 [file Table_1.docx]

**Detailed search strategy for each database**

**PubMed**

#1 "Geriatric Nutritional Risk Index"[Mesh] OR "geriatric nutritional risk index"[tiab] OR "geriatric nutrition risk index"[tiab] OR GNRI[tiab] OR "nutritional risk index"[tiab] OR "nutritional indices"[tiab] OR malnutrition[tiab]

#2 "Transcatheter Aortic Valve Replacement"[Mesh] OR "transcatheter aortic valve implantation"[tiab] OR "TAVI"[tiab] OR "transcatheter aortic valve replacement"[tiab] OR TAVR[tiab]

#3 #1 AND #2

Limits: Humans; From inception to September 22, 2025

**Embase**

('geriatric nutritional risk index'/exp OR 'geriatric nutritional risk index':ti,ab OR 'geriatric nutrition risk index':ti,ab OR gnri:ti,ab OR 'nutritional risk index':ti,ab OR 'nutritional indices':ti,ab OR malnutrition:ti,ab) AND ('transcatheter aortic valve implantation'/exp OR 'transcatheter aortic valve implantation':ti,ab OR 'transcatheter aortic valve replacement':ti,ab OR tavi:ti,ab OR tavr:ti,ab)

Limits: Human studies; Publication date: inception to 2025-09-22

**Web of Science**

TS=("geriatric nutritional risk index" OR "geriatric nutrition risk index" OR GNRI OR "nutritional risk index" OR "nutritional indices" OR malnutrition) AND TS=("transcatheter aortic valve implantation" OR TAVI OR "transcatheter aortic valve replacement" OR TAVR)

Timespan: All years – 2025-09-22

Indexes: SCI-EXPANDED, SSCI, A&HCI, ESCI

Language: No filter (apply English filter manually if needed)
